# Unstabilized DNA breaks in HTLV-1 Tax expressing cells correlate with functional targeting of Ku80, not PKcs, XRCC4, or H2AX

**DOI:** 10.1186/2045-3701-2-15

**Published:** 2012-04-27

**Authors:** Franca Majone, Kuan-Teh Jeang

**Affiliations:** 1 Department of Biology, Via Bassi 58/b, 35131, Padua, Italy; 2 Laboratory of Molecular Microbiology, NIAID, NIH, Bethesda, MD, 20892-0460, USA

**Keywords:** HTLV-1, Tax, Ku80, PKcs, XRCC4, H2AX, DNA damage

## Abstract

**Background:**

Expression of the human T-cell leukemia virus type 1 (HTLV-1) Tax oncoprotein rapidily induces a significant increase of micronuclei (MN) and unstabilized DNA breaks in cells. Unstabilized DNA breaks can have free 3′-OH ends accessible to *in situ* addition of digoxygenin (DIG)-labeled dUTP using terminal deoxynucleotidyl transferase. In the present work, we used a GFP-Tax (green fluorescent protein) plasmid, which produces a functionally active GFP-tagged Tax protein, to detect the cellular target(s) for Tax which might mechanistically explain the clastogenic phenomenon. We examined the induction of MN and unstabilized DNA breaks in wild type cells and cells individually knocked out for Ku80, PKcs, XRCC4, and H2AX proteins. We also assessed in the same cells, the signal strengths produced by DIG-dUTP incorporation at the unstable DNA breaks in the presence and absence of Tax.

**Results:**

Cells mutated for PKcs, XRCC4 and H2AX showed increased frequency of MN and unstabilized DNA breaks in response to the expression of Tax, while cells genetically mutated for Ku80 were refractory to Tax’s induction of these cytogenetic effects. Moreover, by measuring the size of DIG-dUTP incorporation signal, which indicates the extent of unstable DNA ends, we found that Tax induces larger signals than those in control cells. However, in xrs-6 cells deficient for Ku80, this Tax effect was not seen.

**Conclusions:**

The data here demonstrate that clastogenic DNA damage in Tax expressing cells is explained by Tax targeting of Ku80, but not PKcs, XRCC4 or H2AX, which are all proteins directly or indirectly related to the non-homologous end-joining (NHEJ) repair system. Of note, the Ku80 protein plays an important role at the initial stage of the NHEJ repair system, protecting and stabilizing DNA-breaks. Accordingly, HTLV-1 Tax is shown to interfere with a normal cellular protective mechanism for stabilizing DNA breaks. These DNA breaks, unprotected by Ku80, are unstable and are subject to erosion or end-to-end fusion, ultimately leading to additional chromosomal aberrations.

## Background

The HTLV-I Tax oncoprotein induces rapid cytogenetic damage which can be measured by a significant increase in the number of micronuclei (MN) and unstabilized DNA breaks in cells [1-4]. Tax is thought to have both aneuploidogenic and clastogenic effects [3-7]. We previously characterized the phenomenon of Tax-associated clastogenic DNA damage by examining the status of DNA breaks in the nucleus and in the MN in the presence or absence of Tax [4-6]. In particular, we characterized DNA breaks for the presence or absence of free 3′-OH ends [4]. Free 3′-OH ends represent breaks accessible to the *in situ* addition of digoxigenin (DIG)-labeled dUTP using terminal deoxynucleotidyl transferase. On the other hand, an absence of accessible 3′-OH ends suggests that the breaks maybe protected by a protein complex(es).

Unprotected free 3′-OH ends can progress to larger lesions leading to increasingly serious chromosomal defects which may sow the seed for cellular transformation [4-6]. Previously, we were interested to examine the cellular target for Tax in an attempt to explain mechanistically its clastogenic phenomenon. Accordingly, we tested the ability of Tax to induce MN and unstabilized DNA breaks in rodent cells genetically defective for either the Ku80 protein or the catalytic subunit of DNA protein kinase (DNA PKcs) [6]. We found that the Ku80 mutant cells were refractory to the induction of MN by Tax while cells knocked out for DNA PKcs remained responsive to Tax induction of increased MN [6]. Moreover, Tax expression increased the prevalence of unprotected DNA breaks in Ku80-intact cells, but not in Ku80-mutated cells [6], implicating Ku80 as a necessary cellular factor targeted by Tax for engendering clastogenic DNA damage [6].

In the earlier experiments, we studied the frequency of MN and the prevalence of unstable DNA breaks after transfection of an entire cell population with a Tax-expression plasmid, evaluating the bulk cytogenetic damage on all the “transfected” cells without segregating those specifically expressing Tax from those that did not express Tax [1-6]. In the present work, we have focused the analysis to studying the frequency of MN and unstable DNA breaks in cells specifically identified to express GFP-Tax that has been shown in many publications to reflect the activities of wild type Tax protein. Moreover, we have extended the analyses of Tax effects beyond the Ku80 and PKcs proteins [6] to also include the XRCC4 and H2AX proteins. *En toto,* Ku80, PKcs, XRCC4 and H2AX are proteins directly or indirectly involved in NHEJ repair [8-13]. Ku80, PKcs, and XRCC4 function sequentially in the NHEJ pathway which repairs DNA double strand breaks [8,9]. Ku80 substantially protects the breaks [10] allowing subsequent intervention by PKcs [11] which, in turn, appears to be vital for the recruitment of the XRCC4/ligase IV proteins to religate DNA breaks thus completing repair [8,12,13]. The NHEJ system is influenced by the histone H2AX which marks damaged DNA and undergoes various types of modifications in response to double-strand DNA breaks [14,15].

Here, we have employed wild type and Ku80- [16,17], PKcs- [18], XRCC4- [8,19] or H2AX- [20] mutant cells to examine the induction of MN and the prevalence of unstabilized DNA breaks in cells without or with the expression of Tax. In all the cells, DNA breaks were assessed for their frequency and also for their signal strength produced by DIG-dUTP incorporation. To interpret the latter readout, we compared Tax-induced DNA signal-size with corresponding signal-size of breaks induced by etoposide. Etoposide is known to elicit DNA scission [21]. It interferes with the protective action of Ku proteins leaving unstabilized topoisomerase-induced breaks [22]. Because DIG-dUTP signal strength is expected to reflect the size of the DNA lesion at DNA breaks, our approach allows us to quantify the extent of DNA-damage in the various mutant cells by the size of the DIG-dUTP incorporation signals in the presence of Tax or etoposide. Thus, we could compare the relative contribution of loss of Ku80, PKcs, XRCC4, or H2AX to Tax-induced DNA damage.

## Results and discussion

### MN frequencies in hamster or mouse cells expressing GFP or GFP-Tax

**Figure 1:**
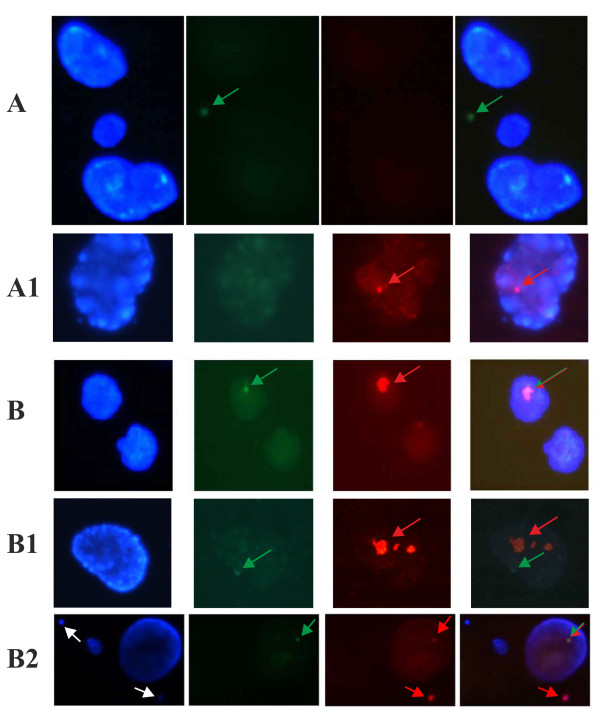
***In situ*DIG-dUTP incorporation in mouse cells transfected with the GFP or GFP-Tax expressing plasmids.**Visualisation of the GFP (green fluorescence, **a)** or GFP-Tax (green fluorescence, b, b1, b2) signals. Counter-staining with DAPI (blue fluorescence). Visualisation of DIG-dUTP incorporation (red fluorescence) in the absence (a1) or in the presence (b, b1, b2) of Tax. *In situ* incorporation was obtained using DIG-dUTP which is incorporated at the free 3-′OH DNA ends and visualised with rhodamine conjugated anti-DIG antibodies. GFP-Tax visualisation traces the presence of Tax (green fluorescence). The Tax signal and the DIG-dUTP incorporation signal in the same nucleus may coincide (b, b2) or may be separate (b1). MN are visible in b2 (white arrows); one MN carries the incorporation signal (red). a, a1, b show H2AX−/− cells; b1, b2 show XRCC4−/− cells. The size of incorporation signal is larger in the presence of Tax (b, b1) than in the absence (a1).

**Figure 2:**
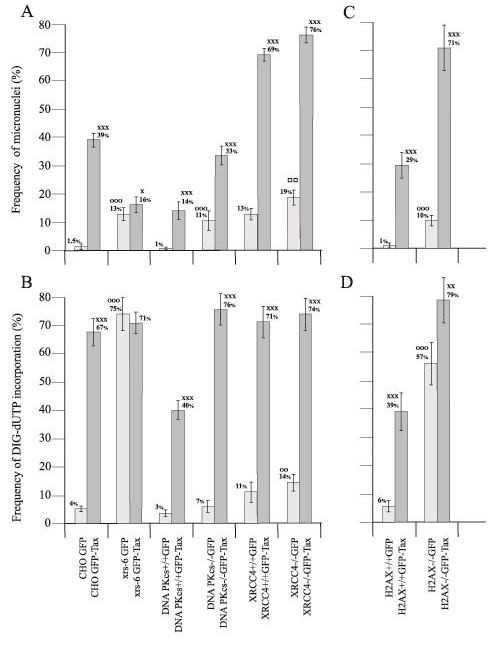
**Cytogenetic effects in hamster and mouse cells. a.**Frequency of micronuclei (MN) in hamster (CHO, xrs-6) and mouse (DNA PKcs+/+, DNA PKcs−/−, XRCC4+/+, XRCC4−/−) cells transfected with the GFP or GFP-Tax expression plasmid**s.** xxx: significantly different (*P* < 0.001) compared to values found in the respective control cells, made up of the same type of cells transfected with the GFP plasmid. x: significantly different (*P* < 0.05) compared to values found in the respective xrs-6 cells transfected with the GFP plasmid. _°°°_: significantly different (*P* < 0.001) compared to values found in the respective wild type control cells transfected with the GFP expression plasmid. □□: significantly different (*P* < 0.01) compared to values found in the respective XRCC4+/+ cells transfected with the GFP plasmid. **b.** Frequency of incorporation of DIG-dUTP in nuclei of hamster (CHO, XRS-6) and mouse (DNA PKcs+/+, DNA PKcs−/−, XRCC4+/+, XRCC4−/−) cells transfected with the GFP or GFP-Tax expression plasmid. xxx: significantly different (*P* < 0.001) compared to values found in the respective control cells, made up of the same type of cells transfected with the GFP plasmid. _°°°_: significantly different (*P* < 0.001) compared to values found in CHO cells transfected with the GFP plasmid. _°°_: significantly different (*P* < 0.01) compared to values found in the XRCC4+/+ cells transfected with the GFP plasmid. **c.** Frequency of MN in H2AX+/+ and H2AX−/− cells showing GFP or GFP-Tax proteins. xxx: significantly different (*P* < 0.001) compared to values found in the respective control cells, transfected with the GFP expression plasmid. _°°°_: significantly different (*P* < 0.001) compared to values found in the respective wild type control cells transfected with the GFP expression plasmid. **d.** Frequency (%) of DIG-dUTP incorporation in nuclei of H2AX+/+ and H2AX−/− cells showing GFP or GFP-Tax proteins. xxx: significantly different (*P* < 0.001) compared to values found in H2AX+/+ cells, transfected with the GFP plasmid. xx: significantly different (*P* < 0.01) compared to values found in H2AX−/− cells transfected with the GFP plasmid. _°°°_: significantly different (*P* < 0.001) compared to values found in H2AX+/+ cells transfected with the GFP plasmid.

To examine the effect of Tax in cells, we transfected cells with expression plasmids for GFP-alone (green fluorescent protein) or GFP-Tax (green fluorescent protein–Tax) [7]. In Chinese Hamster Ovary (CHO) cells transfected with GFP-alone, we observed green signals localized in the cytoplasm (Figure 1a). In GFP-Tax transfections, we identified cells that have green nuclear signals consistent with the expected expression of GFP-Tax fusion protein in the nucleus (Figure 1b, b1, b2). We quantified in these cells the presence of MNs (Figure 1b2). Figure 2a shows MN frequencies in CHO (wild type), xrs-6 (CHO cells lost for Ku80 activity), PKcs+/+, PKcs−/−, XRCC4+/+, and XRCC4−/− cells after transfection with a GFP or GFP-Tax expression plasmid. As seen in Figure 2a, CHO cells expressing GFP have an MN frequency of 1.5% while CHO cells transfected with GFP-Tax have an MN frequency of 39%. By contrast, xrs-6 cells expressing GFP-Tax have an MN frequency of around 16%, and this value is statistically indifferent from the MN frequency (13%) of xrs-6 cells transfected with GFP-alone (Figure 2a).

We next transfected PKcs+/+ and PKcs−/− cells with GFP or GFP-Tax. In the absence of Tax, DNA PKcs−/− cells (11%) have a constitutively higher frequency of MN when compared to DNA PKcs+/+ cells (1%) (Figure 2a). This indicates that loss of PKcs function perturbs normal genome homeostasis and induces MN. In GFP-Tax expressing cells, the MN frequency of PKcs+/+ and PKcs−/− cells increased to around 14% and 33%, respectively (Figure 2a). Thus Tax expression further increases MN frequency in cells wild type for or mutated for PKcs function.

We also compared XRCC4 WT and XRCC4−/− cells. Ambiently, XRCC4−/− cells (19%) show a greater MN prevalence than XRCC4+/+ cells (13%). Thus, the loss of XRCC4 function, which acts at the last step of DNA break repair [8], appears to also make cells more prone to MN. We should note that both XRCC4+/+ and XRCC4−/− cells employed here are p53−/− [8]. Nonetheless, when Tax was expressed in the cells, the observed MN frequencies increased in a statistically significant manner in both XRCC4+/+ (69%) and XRCC4−/− (76%) cells (Figure 2a). Indeed, similar to the loss of PKcs-function, but unlike the loss of Ku80, the loss of XRCC4 activity does not prevent increased MN induction by Tax.

### Frequency of DIG-dUTP incorporation in hamster and mouse cells expressing GFP or GFP-Tax protein

We next evaluated the frequency of DIG-dUTP incorporation in the nuclei of hamster and mouse cells transfected with a GFP or GFP-Tax plasmid. Cells were assayed for DNA breaks by assessing their *in situ* incorporation of DIG-dUTP (red signals; Figure 1b, b1, b2). In Figure 2b, by comparing GFP to GFP-Tax expression in wild type CHO, xrs-6, DNAPKcs+/+, DNAPKcs−/−, XRCC4+/+ and XRCC4−/−, we found that all cells showed statistically significant increases in DIG-dUTP incorporation in GFP-Tax cells compared to GFP-alone cells, with the sole exception of xrs-6 cells. One interpretation of these results is that Ku80 is targeted by Tax in CHO, DNAPKcs+/+, DNA PKcs−/−, XRCC4+/+ and XRCC4−/− cells. By targeting Ku80, Tax increases the amount of incorporation-accessible DNA-damage in these cells. Because xrs-6 cells are already lost for Ku80, Tax-expression in these cells is unable to further increase DIG-dUTP incorporation (Figure 2b). Consistent with this interpretation, it has been found that Tax reduces the expression of Ku80 in cells transformed by HTLV-1 [6,23].

### MN frequencies and DIG-dUTP incorporation in H2AX+/+ and H2AX−/− cells expressing GFP or GFP-Tax protein

H2AX is a histone which facilitates the recruitment of NHEJ repair proteins to DNA breaks [14,15]. Thus, it is indirectly involved in the NHEJ repair system. We further analysed the frequency of MN and *in situ* incorporation of DIG-dUTP in histone H2AX gene knock-out mouse fibroblasts. We transfected paired H2AX+/+ and H2AX−/− cells with a GFP or GFP-Tax plasmid (Figure 1a, b). Figure 2 c shows that the MN frequency in H2AX+/+ cells transfected with the GFP expression plasmid was around 1%, while in the same cells transfected with GFP-Tax, this frequency reached a value of 29%. H2AX−/− cells transfected with GFP (Figure 1a) showed an MN frequency of 10% (Figure 2c), which was significantly higher than GFP-transfected H2AX+/+ cells. In H2AX−/− cells transfected with GFP-Tax, the MN frequency increased to 71% (Figure 2c).

Figure 2d shows that H2AX−/− cells (57%) transfected with GFP-alone have a DIG-dUTP incorporation baseline-frequency significantly higher than that in H2AX+/+ (6%) cells, indicating that the absence of H2AX affects the ambient status of DNA breaks. However, transfection with GFP-Tax significantly increased DIG-dUTP incorporation frequency in both H2AX+/+ and H2AX−/− cells (Figure 2d) suggesting that loss of H2AX does not prevent Tax induced increase in DIG-dUTP incorporation.

### Measuring the size of *in situ* incorporation signals

Beyond the frequency of detecting DIG-dUTP incorporation signals, we additionally evaluated the size of the *in situ* DIG-dUTP incorporation signals under the different experimental conditions. We reasoned that the strength of *in situ* incorporation signal relates to the size of the DNA break. If a discrete break is present in the middle of the DNA and if there is no extension of the break by DNA degradation, then the *in situ* incorporation can be quite small; but, if extended DNA degradation occurs, then greater *in situ* incorporation with a larger signal is expected. This is also the case if a DNA deletion/break happens at the end of a chromosome.

Topoisomerase inhibitors (e.g. etoposide) prevent the rejoining of topoisomerase-induced DNA breaks [22]. Topoisomerase II-induced breaks need Ku proteins for repair [22], and etoposide has been found to interfere specifically with Ku-mediated protection of the topoisomerase-induced breaks. Etoposide has also been associated with increased degradation of DNA [21]; this is perhaps because unprotected breaks are unstable and prone to being degraded. Indeed, we found that the frequency of *in situ* DIG-dUTP incorporation increases significantly in mouse embryo fibroblasts (MEF) treated for three hours with 5 or 10 μg/ml of etoposide (data not shown). Moreover, the incorporation signal was significantly greater in MEFs treated with etoposide compared to control cells (Lines 1, 2, 3; Table 1 and Table 2). We further evaluated the average sizes of the DIG-dUTP incorporation signal in CHO, xrs-6, PKcs+/+, PKcs-/-, XRCC4+/+, XRCC4 −/−, H2AX+/+ and H2AX−/− cells after transfection with GFP or GFP-Tax. For CHO cells, the signal size in the presence (3.81) of Tax was significantly greater than in the absence (1.16) of Tax (Table 1 and Table 2). For PKcs+/+ and PKcs−/− cells, Tax expression produced signal sizes which were significantly greater than cells that did not express Tax (Table 1 and Table 2). In xrs-6 cells, which are absent for Ku80, there was a significantly higher mean basal signal size than in wild type CHO cells, indicating that without Ku80 the size of ambient DNA breaks is generally larger. Unlike Tax expression in wild type CHO which increased DNA break size from 1.16 to 3.81, Tax expression in xrs-6 failed to significantly increase the DIG-dUTP incorporation size (Lines 6, 7; Table 1 and Table 2).

**Table 1:** Mean size (μm) of DIG-dUTP incorporation signals in nuclei of hamster and mouse cells in the presence or absence of Tax or etoposide

**Experimental groups**		**n**	**mean size**	**Standard deviation**
1	Mouse embryo fibroblasts (MEFs)	68	1.37	0.46
2	MEFs + etoposide 5 μg/ml *	46	1.77	1.15
3	MEFs + etoposide 10 μg/ml *	50	2.06	0.96
4	CHO	66	1.16	0,31
5	CHO + Tax	50	3.81	1.74
6	xrs-6	56	1.99	0.82
7	xrs-6 + Tax	120	2.08	0.67
8	PKcs+/+	38	1.43	0.51
9	PKcs+/+ + Tax	70	2.28	1.05
10	PKcs−/−	98	1.35	0.45
11	PKcs−/− + Tax	150	2.35	0.99
12	XRCC4+/+	77	1.63	0.69
13	XRCC4+/+ + Tax	50	3.53	1.68
14	XRCC4−/−	175	1.74	0.67
15	XRCC4−/− + Tax	48	3.39	1.12
16	H2AX+/+	68	1.37	0.46
17	H2AX+/+ + Tax	70	2.50	0.95
18	H2AX−/−	65	2.01	0.76
19	H2AX−/− + Tax	68	3.20	0.90

* = three hours of treatment.

n = number of measured DIG-dUTP incorporation signals.

**Table 2:** Statistical comparison (Anova)

1 vs. 2	●●	6 vs. 7	ns	12 vs. 14	ns
1 vs. 3	●●●	8 vs. 9	●●●	14 vs. 15	●●●
2 vs. 3	ns	8 vs. 10	ns	16 vs. 17	●●●
4 vs. 5	●●●	10 vs. 11	●●●	16 vs. 18	●●●
4 vs. 6	●●●	12 vs. 13	●●●	18 vs. 19	●●●

ns = not significantly different; significantly different: ●● = *P* < 0. 01; ●●● = *P* < 0. 001.

Basally, XRCC4+/+ and XRCC4−/− cells were not significantly different for *in situ* incorporation signal sizes (Lines 12, 14; Table 1 and Table 2). The size of the DIG-dUTP signal, however, increased significantly in both XRCC4+/+ and XRCC4−/− cells with the expression Tax (compare line 12 to line 13; line 14 to line 15; Table 1 and Table 2). On the other hand, the size of the basal DIG-dUTP incorporation signal was significantly higher in H2AX−/− cells compared to H2AX+/+ cells (compare line 18 to 16; Table 1 and Table 2) indicating the important contribution of H2AX histone to DNA break stability. The expression of Tax further increased the measured break sizes in H2AX+/+ and in H2AX−/− cells (Lines 17, 19; Table 1 and Table 2). We note that, with the exception of xrs-6, all other cells in Table 1 and Table 2 have wild type Ku80. Our collective results indicate that cells intact for Ku80, but not cells without Ku80, show Tax increased DIG-dUTP signal sizes. This finding supports the interpretation that the DNA break-protection activity of Ku80 is functionally targeted by Tax.

## Conclusions

Results in this study confirm that the clastogenic DNA damage in HTLV-1 Tax-expressing cells is in part explained by Tax targeting of the Ku80 subunit of the Ku70/Ku80 heterodimer which play an important role in NHEJ (non homologous end joining) repair [6]. Elsewhere, we have also reported that Tax can directly cause DNA damage through the induction of reactive oxygen species [24]. One interpretation of our current data is that Tax suppresses Ku80 -recognition and -stabilisation of damaged DNA ends. The DNA ends left unprotected are unstable and are subject to erosion or end-to-end fusion, producing additional chromosomal aberrations. These conclusions are supported by findings in the hamster xrs-6 cells genetically mutated for Ku80 wherein neither the frequency of MN nor of unstable DNA breaks is increased by Tax. By contrast, in primary murine DNAPKcs −/− cells, which is knocked out for PKcs but intact for Ku80, Tax significantly increased both the frequency of MN and DNA breaks. Taken together, the experimental results indicate that Tax’s interference with the NHEJ repair pathway is at the level of Ku80, not at PKcs. Additional data in this study also show that Tax does not significantly target XRCC4 or H2AX.

By measuring the strength of DIG-dUTP incorporation which correlates with the size of DNA breaks [4,6,23], we found that Tax expression generally induced higher DIG-dUTP signals. We suggest that Tax targets Ku80 leaving DNA breaks unprotected making them susceptible to additional degradation thereby increasing the incorporation of DIG-dUTP. Again, in xrs-6 cells deficient for Ku80, this Tax targeting of Ku80 cannot occur; thus there is no further Tax-increased DIG-dUTP signal incorporation. We note that a consequence of destabilisation of DNA breaks is increased DNA end-to-end fusions [25]. End to end fusions, which may serve to contribute to cellular transformation, have indeed been observed in the presence of Tax [5].

Ku80 joins a long list of cellular proteins that are targeted by Tax which include checkpoint proteins such as p53, Mad1, CHK1/2 and proproliferative factors like, AKT, NF-kB, cyclins and cyclin dependent kinases [26-30]. Future investigations will help shed additional light on how these factors interact functionally in the course of HTLV-1 engendered ATL development.

## Materials and methods

### Cells and transfection

Hamster xrs-6 (genetically mutated for Ku80) cells [16], CHO wild type cells, mouse embryonic fibroblasts (MEFs) lacking either the PKcs [18] or the XRCC4 [8] or the H2AX [20] proteins and the respective parental strains (XRCC4 cells are p53−/−) were cultured as monolayers in Dulbecco’s Modified Eagle Medium (DMEM) supplemented with 10% fetal bovine serum (FBS) (Gibco-Invitrogen, Carlsbad, CA). Where indicated, cells were transiently transfected using calcium phosphate with a GFP or GFP-Tax expression plasmid [7]. The cells were surveyed 48 hours later for cytogenetic effects.

### Micronuclei (MN) assay and fluorescence *in situ* incorporation

Interphase preparations for MN assay and fluorescence *in situ* incorporation were obtained following the procedures previously described [1-4,6,23]. Fluorescence *in situ* incorporation was carried out using terminal deoxy-nucleotidyl transferase (TdT) (Roche Applied Science, Indianapolis, IN), which catalyses the addition of deoxyribonucleotide triphosphates to the 3′-OH DNA ends. To the substrates of TdT, Digoxigenin-11-dUTP (Dig-dUTP) (Millipore,Billerica,MA) was added to the 3′-OH ends. Antibody detection of DIG-dUTP labelling employed a specific antibody linked to rhodamine (Millipore,Billerica,MA). The experimental protocol was performed following the procedure previously described [4,6,23]. To determine the frequencies of MN and unstabilized DNA breaks in nuclei, for each experimental point, 500 GFP or GFP-Tax expressing cells were counted. For each experimental point three independent experiments were performed, and pooled data from these experiments were considered for statistical analysis. Differences between data from spontaneous and Tax induced cytogenetics effects were tested for significance using the G test [31].

### Characterization of unstabilized DNA breaks and scoring of the slides

To characterize unstabilized DNA breaks, we measured the size of *in situ* DIG-dUTP incorporation signal (green signal) in hamster and mouse cells in the presence or absence of Tax; in particular, we calculated the length of major axis of the DIG d-UTP incorporation signal. All the analyses were performed on a LEICA (5000B) microscope with LAS software (using also a measure module); digital camera was LEICA DFC426C. Differences between measures of the DIG-dUTP incorporation signals in the different experimental conditions were tested for significance using analysis of variance (ANOVA).

## Abbreviations

HTLV-1 = Human T-cell leukemia virus type 1; MN = Micronuclei.

## Competing interests

The authors declare that they have no competing interests.

## Authors’ contributions

FM and KTJ conceived and designed the experiments and wrote the manuscript. FM performed all the experiments in the manuscript. All authors read and approved the final manuscript.
